# Anger Emotional Stress Influences VEGF/VEGFR2 and Its Induced PI3K/AKT/mTOR Signaling Pathway

**DOI:** 10.1155/2016/4129015

**Published:** 2016-02-14

**Authors:** Peng Sun, Sheng Wei, Xia Wei, Jieqiong Wang, Yuanyuan Zhang, Mingqi Qiao, Jibiao Wu

**Affiliations:** ^1^College of Pharmacy, Shandong University of Traditional Chinese Medicine, Jinan, Shandong 250355, China; ^2^Experiment Center, Shandong University of Traditional Chinese Medicine, Jinan, Shandong 250355, China; ^3^Technical Office of Pharmacology, Shandong Institute for Food and Drug Control, Jinan 250351, China; ^4^School of Preclinical Medicine, Shandong University of Traditional Chinese Medicine, Jinan, Shandong 250355, China

## Abstract

*Objective.* We discuss the influence of anger emotional stress upon VEGF/VEGFR2 and its induced PI3K/AKT/mTOR signal pathway.* Methods.* We created a rat model of induced anger (anger-out and anger-in) emotional response using social isolation and resident-intruder paradigms and assessed changes in hippocampus' VEGF content, neuroplasticity, and the PI3K/AKT/mTOR signaling pathway.* Results.* The resident-intruder method successfully generated anger-out and anger-in models that differed significantly in composite aggression score, aggression incubation, open field behavior, sucrose preference, and weight gain. Anger emotional stress decreased synaptic connections and VEGFR2 expression. Anger emotional stress led to abnormal expression of VEGF/VEGFR2 mRNA and protein and disorderly expression of key factors in the PI3K/AKT/mTOR signal pathway. Fluoxetine administration ameliorated behavioral abnormalities and damage to hippocampal neurons caused by anger emotional stress, as well as abnormal expression of some proteins in VEGF/VEGFR2 and its induced PI3K/AKT/mTOR signal pathway.* Conclusion.* This research provides a detailed classification of anger emotion and verifies its influence upon VEGF and the VEGF-induced signaling pathway, thus providing circumstantial evidence of mechanisms by which anger emotion damages neurogenesis. As VEGFR2 can promote neurogenesis and vasculogenesis in the hippocampus and frontal lobe, these results suggest that anger emotional stress can result in decreased neurogenesis.

## 1. Introduction

Research has shown that anger is one of the negative, most influential emotional responses in interpersonal relationships and social harmony; it is also the most stressful response and is comorbid with pathogenesis. Anger is closely linked with cardiovascular and cerebrovascular diseases, digestive system diseases, and cancer, as well as psychological disorders and related behaviors, such as depression and suicide. As anger serves as a disease-inducing factor, it is important to study the biological foundation of this emotion [1, 2].

At present, the state of research on the biological mechanism of anger emotion is relatively limited. As the core expression of anger, aggressive behavior has been used as a measure of anger across the literature. The substrate of aggressive action is a physiological cascade that involves GABA and 5-HT, as well as neurosteroids [3–5]. This results in overactivation of the amygdaloid nucleus, which is in charge of impulse control, causing the prefrontal cortex to malfunction [6–11]. Hormones also play an important role in human aggression. Moderate levels of progesterone, high levels of testosterone, and a lowered level of corticosterone appear to be key factors mediating aggressiveness [12–16]. Furthermore, allosteric modification of GABA_A_R is closely related to aggressive behavior. Moderate levels of allopregnanolone, high levels of androstenediol, and a positive endogenous modifier of GABA_A_R can increase aggressiveness [17–20]. Decreased 5-HT can also lead to aggressive behavior. Decreased expression of 5-HT_1A_ receptors in the prefrontal cortex and increased raphe nucleus density are also relevant to aggression [21].

Contemporary research focuses on the cellular and molecular foundation of mental diseases on trophic factors, with relevant cell types covering neurons and endothelial cells. These cells, with highly varied functions, interact with each other through common signaling molecules. Disorders of cell signaling pathways can influence cerebral function and behavioral expression. Multifunction trophic factors, such as vascular endothelial growth factor (VEGF), can ameliorate defects in vessel and nerve growth to affect emotional expression and psychodynamic cognition processes. Understanding the behavior and cellular and molecular function of multifunction trophic factors like VEGF may elucidate novel therapeutic targets [22]. VEGF also promotes angiogenesis, vascular endothelial growth, and vascular permeability.

In recent years, VEGF has been found to promote hippocampal neuron growth. Research has confirmed that this VEGF-induced stimulation of neurogenesis plays an important role in neuropsychological diseases and stress responses. VEGF participates in the stress response to relevant diseases, particularly depression, and it also plays a role in the activity of antidepression drugs through serotonin selective reuptake inhibitor (SSRI) and interacts with the 5-HT system [23]. We predict that VEGF may participate in the expression of anger emotion caused by a psychological stress response. We investigated the influence of this process on VEGF and the possible regulating mechanisms. An understanding of the influence of anger emotion stress response abnormality upon VEGF and its regulating processes may inform the biological foundation of anger emotion.

The current research was designed to create rat models of anger-out and anger-in expression induced by the resident-intruder paradigm. By measuring the compound influence of stress response and fluoxetine intervention upon aggressive behavior, open field behavior, and sucrose preference level, the behavioral features of anger-out and anger-in rats with different responses induced by the resident-intruder paradigm were revealed. We used electron microscopy to observe the microstructure of hippocampal synapse of model rats and explore the influence of anger emotional stress upon the microstructure morphology of hippocampus; immunofluorescent staining was used to further probe the influence of anger emotional stress upon neuron expression of VEGFR2. Real-time PCR and Western blot assays were used to analyze mRNA and protein expression of hippocampal VEGF and VEGFR2, as well as the signal pathway changes of PI3K/AKT/mTOR induced by VEGFR2, in order to explore relevant biological foundation of VEGF in anger emotional stress expression. Our research further revealed the function of VEGF, with anger as the chronic psychological stress damage disease-inducing factor, and the function of VEGF and the relevant signal pathway in relation to psychological stress damage. Taken together, these results may provide a scientific foundation for researching and developing intervening drugs for treating anger-related pathology.

## 2. Materials and Methods

### 2.1. Experiment Animals and Drugs

SPF-grade healthy Wistar rats, weighing 250–270 g, and SD rats, weighing 180–200 g, were used (production permit number SCXK (Beijing) 2012-0001, Beijing Vital River Experiment Animal Co., Ltd.). Wistar rats were used as experimental animals and were randomly divided into groups. SD male rats were designated as intruders, eliciting the emotion and behavior of experiment rats. Each group of experimental rats was weighed at key time points during the study to assess weight gain.

Laboratory animals were provided by the* Laboratory Animal Center of Shandong Traditional Chinese Medicine University* (license number: SCXK (LU) 2011-0003). Laboratory animals were cared for according to “The Care and Use of Laboratory Animals” by the* Laboratory Animal Center of Shandong Traditional Chinese Medicine University*.

Fluoxetine capsules were used (Suzhou Chung-Hwa Pharmaceutical Co., Ltd., approval number: 53513004). Based on clinical equivalence conversion, an intragastric dose of 0.267 mg/100 g/d was administered to rats according to product manual.

### 2.2. Macro Behavioral Experiment

#### 2.2.1. Open Field Test

An open field test chamber, made of opaque materials and measuring 100 cm × 100 cm × 40 cm, was used. The inside wall and floor were gray, and the bottom was divided into 25 equal zones. The entire field was recorded from above using a camera. During the test, each rat was gently placed into the middle grid of the open field chamber, and its general movement path during 3 min was recorded (Panlab Smart-15 software). The experiment was conducted in a quiet environment. To account for circadian movement changes, all studies were conducted at the same time of day. Before each experiment, ethyl alcohol was used to clean the floor of the chamber [24, 25].

#### 2.2.2. Sucrose Preference Experiment

The sucrose preference experiment [26, 27] included 48 h sucrose drinking training and 1 h test after 24 h of training. 48 h before the experiment, rats were habituated to drinking water with sucrose. Two bottles of water, with equivalent weights, were given to animals, one containing 1% sucrose solution and the other containing common tap water. Every 12 h, the position of the two bottles was shifted so as to avoid the positional effect of rats in drinking behavior. After the training, rats were provided with tap water for 6 h. After 18 h of providing no water or food, each rat was given two bottles of water (one containing 1% sucrose solution and the other containing common tap water) for them to drink for 1 h. Consumption of each liquid was calculated from the weights of the bottles. Sucrose preference was calculated as follows: sucrose preference rate = sucrose consumption (g)/[sucrose consumption (g) + water consumption (g)] × 100%.

#### 2.2.3. Resident-Intruder Anger Emotional Stress Model and Its Subtype

Animals were placed on a reverse light cycle to habituate to the environment for one week and bred in isolation for one more week. Then, they were sent into the resident-intruder experiment. After one week in resident-intruder experiment, the score of composite aggressive behavior was calculated. Experimental rats were divided into two groups based on the median method as follows: High Aggressive (HA) described the anger-out group, while Low Aggressive (LA) described the anger-in group [28]. Composite aggression was calculated using the following formula: composite aggression = [(number of attacks) + 0.2 × (attack duration) + (number of bites) + 0.2 × (on-top duration) + (piloerection)].

### 2.3. Transmission Electron Microscopy (TEM)

On the day following drug administration, 40 mg/kg pentobarbital sodium (Sigma-Aldrich) was used for intraperitoneal anesthesia. The specific steps were as follows: opened the chest to expose the heart, inserted the tube through ascending aorta of the left ventricle, and then used scissors to cut open the auricula dextra; used precooled 0.9% NaCl solution (provided by Qilu Hospital, Jinan) followed by 4°C 4% paraformaldehyde, 0.1 mol/L PBS (Beyotime Institute of Biotechnology, pH7.4) for perfusion, then opened the skull after hardening to take out the cerebral tissues to be fixed with 2.5% glutaraldehyde (Sigma-Aldrich). In reference to the stereotaxic atlas of brain, took 3 parts of brain tissues, measuring 2 mm × 2 mm × 2 mm, out of hippocampus to be rinsed 8 times for a duration of 48 h with 0.1 M phosphate buffer; used 2% osmic acid (Sigma-Aldrich) to fix for 1.5 h and rinsed with PBS 5 times; dehydrated with 50%, 70%, 80%, 90%, 95%, and 100% acetone I for 10 min and with 100% acetone II and III for 40 min, respectively; used 812 epoxy resin and acetone (Sigma-Aldrich) 1 : 1 mixed liquor for embedding and saturation for 1.5 h. Pure resin was embedded and polymerized for 3 h at room temperature, 12 h at 37°C, 12 h at 45°C, and 48 h at 60°C; trimmed the embedding end; sliced and double stained with uranium lead; and then observed [29] them under TEM (JEOL, JEM-1400).

### 2.4. Fluorescent Immunohistochemistry

Sections of hippocampus and frontal lobes were selected based on the stereotaxic atlas of the rat brain. The distribution of VEGFR2 in lobes and hippocampal brains was observed with a laser scanning confocal microscope (LSM510, ZEISS, Germany). Three different angles were chosen from each target brain area in each slice for imaging [30, 31]. The primary antibody used was anti-VEGFR2 (Abcam, ab131441, dilution ratio: 1 : 1000), and the secondary antibody was IgG marked with FITC (Wuhan Boster Biological Engineering Co., Ltd., BA1105, dilution ratio: 1 : 50).

### 2.5. Real-Time qPCR

After the splitting decomposition of samples, total RNAs (RNA Lyzol, Shanghai ExCell Biology Co., Ltd., MB000-0012) were extracted and reverse transcription (Thermo Scientific RevertAid First Strand cDNA Synthesis Kit, Thermo Scientific Co., Ltd., K1622) was performed according to the manufacturer's instructions. The prepared cDNA samples were used for fluorescent quantification via RT-PCR (Real-time PCR Master Mix, Toyobo Co., Ltd., QPK-101T). There were 3 duplication wells for each sample; 7500 Fast system software was used for analysis [32]. The primer and sequence used were as shown in Table 1.

### 2.6. Western Blotting

The Western blotting experiment was conducted according to standard procedures [33]. The primary antibodies used and their dilution ratios are shown in Table 2. The second antibody was horseradish peroxidase- (HRP-) conjugated goat anti-mouse IgG (Jackson ImmunoResearch, 115-035-166) or HRP-conjugated goat anti-rabbit IgG (Jackson ImmunoResearch, 111-035-144), with a dilution ratio of 1 : 5000.

### 2.7. Statistics

All data were analyzed with GraphPad Prism 5 statistical software and are shown as mean ± SEM. One-way ANOVA was used for analysis and processing, and *P* < 0.05 indicated statistical significance.

## 3. Results

### 3.1. Anger-Out and Anger-In Stress Rat Modeling and Behavior Evaluation

**Figure 1:**
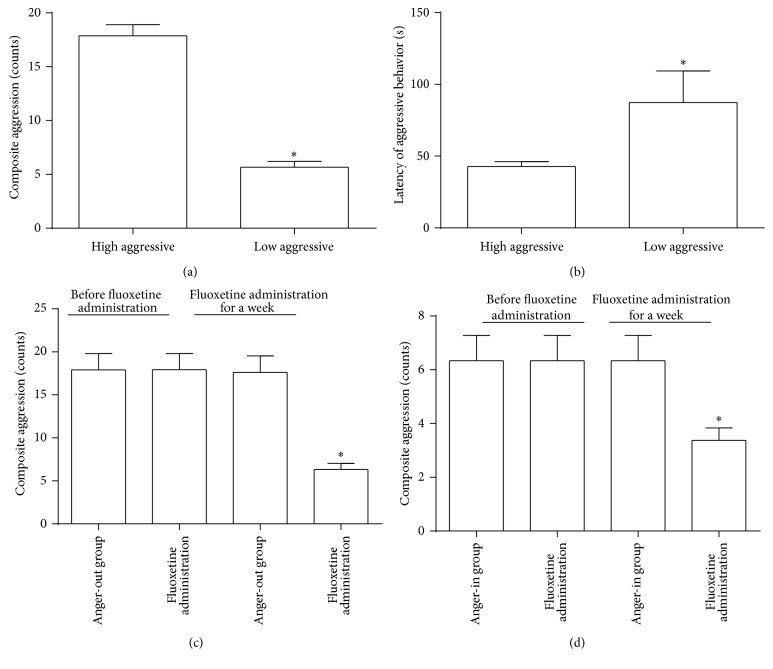
(a) Composite aggression scores of HA group and LA group (*n* = 36, ^*∗*^ *P* < 0.05). (b) Latency of aggressive behavior of HA group and LA group (*n* = 36, ^*∗*^ *P* < 0.05). (c) Composite aggression scores of anger-out group before and after fluoxetine administration (*n* = 12, ^*∗*^ *P* < 0.05). (d) Composite aggression scores of anger-in group before and after fluoxetine administration (*n* = 12, ^*∗*^ *P* < 0.05).

We mainly referred to the anger stress model based on resident-intruder paradigm out of territory awareness proposed by Prof. Sieste F. de Boer. After one week in the resident-intruder mode, the scores of composite aggression were tested and calculated, and the resident rats were divided into two groups according to the median method. HA rats were assigned to the anger-out group (Figure 1(a)) and LA rats were assigned to the anger-in group (Figure 1(a)). We also measured the latency of aggression. The HA group showed shorter latency (Figure 1(b)) and the LA group showed longer latency (Figure 1(b)). Division into HA and LA groups based on the median method of composite aggression score was practically feasible.

We randomly divided the anger-out group, namely, the HA group, into two groups, including anger-out model group and anger-out model group with administration of fluoxetine (Figure 1(c)). We also divided the anger-in group into two groups randomly, namely, anger-in model group and anger-in model group with administration of fluoxetine (Figure 1(d)). It has been shown that fluoxetine can effectively ease depression, anxiety, and anger emotions induced by various factors, so fluoxetine serves as an important tool in studying anger emotion model. After one week of drug administration, fluoxetine was able to effectively cut down the aggression scores of both anger-out and anger-in groups (Figures 1(c) and 1(d)).

**Figure 2:**
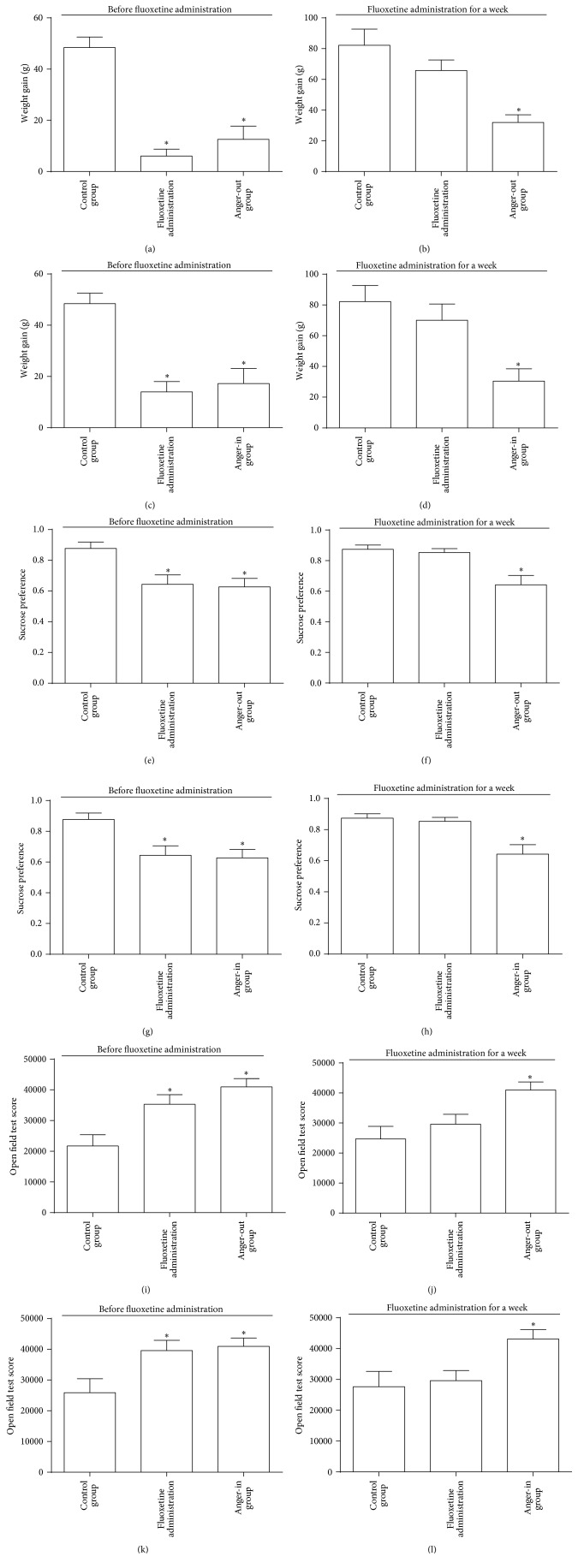
Quantitative analysis (*n* = 12) in weight gain (a, b, c, d), sucrose preference (e, f, g, h), and open field test (i, j, k, l) of anger-out (a, b, e, f, i, j) and anger-in (c, d, g, h, k, l) groups before (a, c, e, g, i, k) and after (b, d, f, h, j, l) fluoxetine administration (^*∗*^ *P* < 0.05).

Before model making, we tested the body weights for all groups as the baseline. The body weights were statistically the same among groups. At key times in modeling, we analyzed weight gain. After one week in the resident-intruder mode, the added weight of each group (including model group and group of fluoxetine administration) significantly decreased when compared with the control group (Figures 2(a) and 2(c)). After one week of drug administration, the added weight of model group still decreased when compared with the control group. Meanwhile, the added weight of the group with drug administration had returned to the level of the control group (Figures 2(b) and 2(d)). It is worth noting that, after one week in the resident-intruder mode, although we conducted statistics and analysis of the weight gains of each group, drug had not been administered. In other words, after two weeks in the resident-intruder mode, the group with drug administration had only received the drug for one week.

We also conducted the sucrose preference test and open field test. The results of the sucrose preference experiment showed that after one week in the resident-intruder mode the sucrose preference level of each group dropped significantly (Figures 2(e) and 2(g)); after one week of drug administration, the sucrose preference level of the model group registered a significant decrease when compared with the control group, while the group with drug administration returned to the level of the control group (Figures 2(f) and 2(h)). On the other hand, open field test results showed that after one week in the resident-intruder mode the open field score for each group increased significantly (Figures 2(i) and 2(k)); after one week of drug administration, the open field score of the model group improved when compared with the control group, while the group with drug administration returned to the level of the control group (Figures 2(j) and 2(l)).

### 3.2. Influence of Anger Stress upon the Microstructure of Hippocampal Synapse of Rats as well as the Distribution and Number of VEGFR2 Positive Cells in Hippocampus and Lobe 

**Figure 3:**
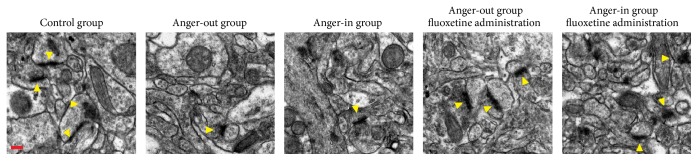
Synaptic ultrastructure in hippocampal CA3 of control, anger-out, anger-in, fluoxetine treated anger-out, and fluoxetine treated anger-in groups. Arrow heads were used to highlight synapses. Scale bar: 0.2 *μ*m.

Hippocampal neurons in the control group were complete and clear in structure, with structurally complete mitochondria, clear mitochondria ridge, full and rich postsynaptic density, and clearly visible synaptic vesicles, as well as increasing synaptic connections (Figure 3). Mitochondrial structure in the model group was normal, with spindle-shaped neurons, increased ribosomes, and decreased numbers of other cells, as well as decreased synaptic connections. Administration of fluoxetine was able to improve these deficits to some extent (Figure 3).

**Figure 4:**
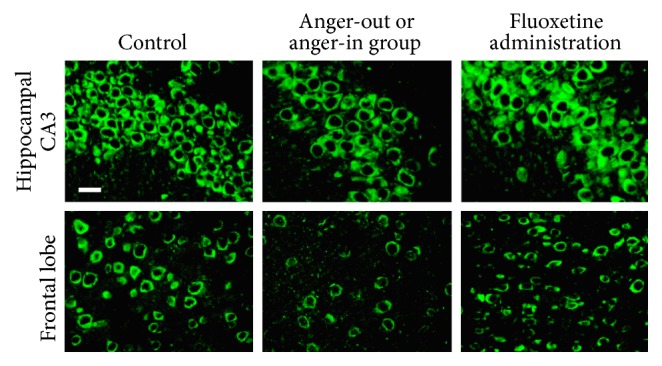
Hippocampal CA3 and frontal lobe (of control, anger-in/anger-out, and fluoxetine administration groups) were stained with anti-VEGFR2 antibody. Scale bar: 100 *μ*m.

Immunofluorescence results showed that there were a large number of VEGFR2 positive cells in the hippocampus and CA3 area, with closely arrayed pyramidal cells (Figure 4). In addition, the number of VEGFR2 positive cells in the hippocampus and CA3 area of rats subjected to the resident-intruder paradigm decreased, with loose array and irregularity (Figure 4). Moreover, both anger-in and anger-out groups exhibited decreased number of VEGFR2 positive cells. These deficits were attenuated after administration of fluoxetine (Figure 4).

### 3.3. Influence of Anger-Out and Anger-In Stress upon VEGF and VEGFR2 in Rat's Hippocampus and Lobe, as well as the Induced PI3k/AKT/mTOR

**Figure 5:**
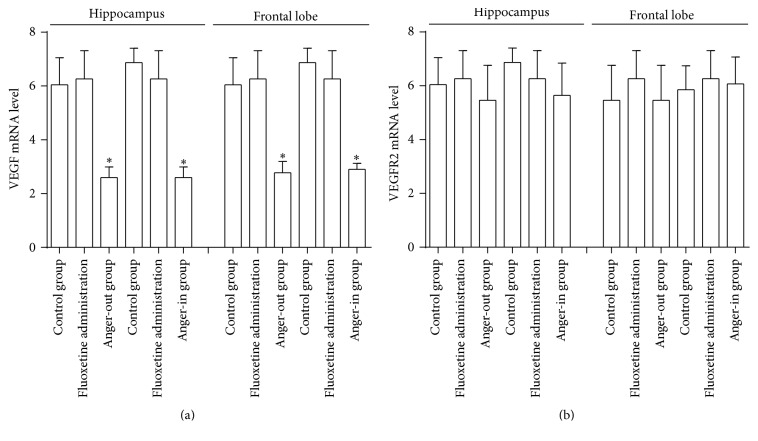
Quantitative analysis (*n* = 5) in VEGF mRNA (a) and VEGFR2 mRNA (b) of hippocampus and frontal lobe. Control, anger-out, anger-in, fluoxetine treated anger-out, and fluoxetine treated anger-in groups were tested (^*∗*^ *P* < 0.05).

We used fluorescent quantitative PCR to detect VEGF and VEGFR2 mRNA levels in rat's hippocampus and frontal lobe. The results showed that, when compared to the control group, the mRNA expression of VEGF in hippocampus and lobe decreased in the model group (Figure 5(a)) and that fluoxetine administration could significantly attenuate decreases caused by anger emotional stress (Figure 5(a)). However, there was no significant difference between the two brain regions of VEGFR2 mRNA in each group (Figure 5(b)).

**Figure 6:**
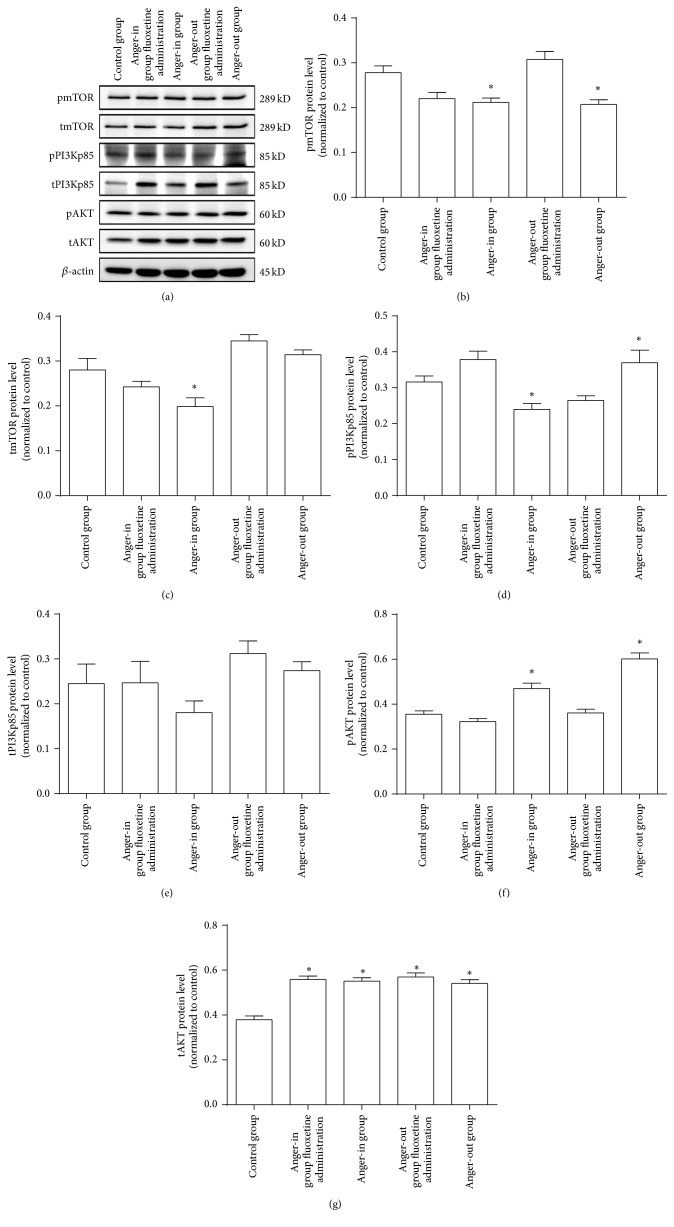
(a) Western blotting was performed with hippocampus' samples of control, anger-out, anger-in, fluoxetine treated anger-out, and fluoxetine treated anger-in groups. (b) Quantitative analysis (*n* = 8) in pmTOR protein level (^*∗*^ *P* < 0.05). (c) Quantitative analysis (*n* = 8) in tmTOR protein level (^*∗*^ *P* < 0.05). (d) Quantitative analysis (*n* = 8) in pPI3Kp85 protein level (^*∗*^ *P* < 0.05). (e) Quantitative analysis (*n* = 8) in tPI3Kp85 protein level. (f) Quantitative analysis (*n* = 8) in pAKT protein level (^*∗*^ *P* < 0.05). (g) Quantitative analysis (*n* = 8) in tAKT protein level (^*∗*^ *P* < 0.05).

We then used Western blotting to detect expression changes of relevant proteins in the PI3k/AKT/mTOR signal pathway induced by VEGF and VEGFR2 (Figure 6(a)). The results showed that the anger-out group registered decreased pmTOR protein expression compared to the control group (Figure 6(b)) and that administration of fluoxetine could improve the situation significantly (Figure 6(b)). When compared with the control group, pmTOR expression in the anger-in group decreased, and fluoxetine administration failed to improve it (Figure 6(b)).

The tmTOR expression of the anger-out group had no significant changes when compared with the other groups, including the control group (Figure 6(c)). The tmTOR expression of the anger-in group decreased significantly when compared with the control group, and fluoxetine administration failed to improve it (Figure 6(c)).

As for pPI3Kp85 level, its expression in the anger-out group registered greater improvement than the control group (Figure 6(d)), and fluoxetine administration greatly improved the expression of pPI3Kp85 level in the anger-out group (Figure 6(d)). The expression of pPI3Kp85 in the anger-in group registered a greater decrease than the control group (Figure 6(d)), and fluoxetine administration could greatly improve the situation (Figure 6(d)). The tPI3Kp85 expression of all experimental groups (including anger-out group, anger-in group, and both fluoxetine administration groups) had no significant changes when compared with control group (Figure 6(e)).

Moreover, the pAKT expression at the anger-out group showed greater improvement than the control group (Figure 6(f)), and fluoxetine administration significantly increased pAKT expression in the anger-out group (Figure 6(f)). The expression of pAKT in the anger-in group registered a greater improvement than the control group, and fluoxetine administration significantly increased pAKT expression among anger-in rats (Figure 6(f)).

Finally, we discovered that tAKT expression in the anger-out group registered a greater improvement than the control group (Figure 6(g)) and that fluoxetine administration failed to improve it (Figure 6(g)). The expression of tAKT in the anger-in group also registered a greater improvement than the control group, and fluoxetine administration had no statistically significant effect in improving the expression (Figure 6(g)).

## 4. Discussion

This research takes advantage of the resident-intruder paradigm to establish the anger emotional model, further divides anger into two groups, anger-out and anger-in, and then explores the biological mechanisms of anger in living creatures. Behavioral changes induced by the resident-intruder paradigm are considered to be similar to human aggression. Although aggressive behavior is not defined as anger, the anger neuroendocrine mechanism is very similar to the human emotion of anger [34] and it is widely believed that aggressive behavior is caused by anger [35].

As a result of population research, scientists have divided anger into anger-out and anger-in groups. Twice, they have developed and revised the state-trait anger inventory (STAXI) to distinguish anger into two different modes of expression [36]. Present research distinguishes anger-out group from anger-in group according to the median method of aggression scores. After one week of the resident-intruder mode, we observed decreased weight gain in each group when compared with the control group. This demonstrates the same effect of inhibiting weight gain through emotional stress, as observed with chronic unpredictable mild stress (CUMS) [37]. After one week of fluoxetine administration (second week of resident-intruder paradigm), there was no significant difference of weight gain between anger emotional stress groups and the control group. On the other hand, after one week in the resident-intruder mode, the anger-out group had a higher score in composite aggression than the control group, and anger-out group exhibited a shorter latency of aggression than the anger-in group. After fluoxetine administration, the score of composite aggression in each anger emotional stress group dropped significantly, compared to groups without administration. The above-described information indicates that the modeling is successful in setting up the major evaluation indexes.

Rodents are very sensitive to sweet solutions (common sugar water), so sucrose drinking amount and sucrose preference level are often used to evaluate the degree of anhedonia. They are more frequently used in depression and anxiety models and serve as an important sensitive index for evaluation [38]. This is the first study to link anhedonia to anger emotion. After one week in the resident-intruder mode, the sucrose preference level significantly decreased at each group when compared with the control group, but after fluoxetine administration the level of the model group still dropped greatly, while the level at the group with administration returned to the level of the control group, revealing the presence of anhedonia during expression of the emotion of anger. The open field test is designed to assess cognition of, anxiety-like behavior towards, and interest in the outside world and assesses behavioral inhibition and increased fear, as well as decreased social behavior, through the activity level of subjects [39]. The open field behavior can be measured using several parameters, among which horizontal score reflects the excitement of animals and vertical score reflects the uncertainty and exploration trend of animals against the surroundings. The total score in open field test is a general reflection of the exploratory behaviors and motivation of animals. After one week in the resident-intruder model, the total score of open field test of each group improved significantly when compared with the control group; fluoxetine administration produced control-like behavior in the open field test. This indicates that the motivation of rats was increased following the resident-intruder test and that fluoxetine was able to regulate this emotionality.

Although aggressive behavior is not defined as anger, this research modeled valid subtypes for anger based on composite aggression scores and obtained valid results from subsequent behavioral tests. As a result, we believe that aggressive behavior plays an important role in the generation and development of anger emotion, which, to some degree, prompts us to connect the research on anger and the research on aggression. Research shows that disorders of the 5-HT system can lead to impulsive aggressive behavior [40–44]. In addition, activation and distribution of different 5-HT receptors are related to aggression [45]. 5-HT1A receptors are distributed in presynaptic membranes of the raphe nucleus, prefrontal cortex, and amygdala and postsynaptic membranes of 5-HT neurons in the hippocampus. Our work shows that rats with anger stress had more spindle-shaped hippocampal neurons, more ribosomes, less organelles, and fewer synaptic connections (Figure 3) and that, after fluoxetine administration, these deficits could be improved to some extent (Figure 3). These results provide evidence for a relationship between aggression and anger.

Synaptic plasticity serves as important means for message transfer and processing among neurons. Here we show evidence of damage to microstructures in the hippocampal CA3 area among anger-out and anger-in rats. The decrease in synapse number and structural damage can influence the release of neurotransmitters, hence hindering message transfer in the central nervous system. The damage to the neurons in the hippocampal CA3 area and microstructure of synapse indicates that anger emotional stress harms the plasticity of central nervous system. Meanwhile, chronic stress, caused by emotional stress exceeding the bearing capacity of the living body, will cause disorders in many aspects like mood, nerve, internal secretion and immunity, and changes in brain structure, as well as learning and memory.

There have been multiple studies concerning the role of VEGF in depression, with a focus on the PI3K/AKT/mTOR signal pathway influencing the process of pathogenesis [46] and neurogenesis [47]. As emotional stress abnormality, after discovering the influence of anger emotion upon hippocampal structure, this research further explored the effects of anger on the PI3K/AKT/mTOR signal pathway induced by VEGF and its receptor VEGFR2. It was found out that the content of hippocampal VEGF dropped during anger emotional stress. The expression of mRNA of VEGF in hippocampus and lobes decreased, while there was no significant change to the mRNA expression of VEGFR2. The reason may lie in the fact that VEGFR2 plays its role mainly through protein expression levels. The mode of anger expression not only is connected with external stress but also is related to individual physiological and psychological features, which determines different responses of organisms to external stress. The expression level of key proteins in PI3K/AKT/mTOR signaling pathway is in disorder, pmTOR, pPI3Kp85, and pAKT in particular. Protein phosphorylation is called the “door to life,” as a protein will be activated after phosphorylation, giving way to its biological function. This complexity of change may be related to the complexity and networking nature of signal pathway. Further research will be required to assess these effects.

In summary, this research provides a detailed classification of anger emotion and provides evidence of the influence of anger emotion upon VEGF and its signal pathway. This may provide a mechanism by which anger impairs neurogenesis through influencing VEGF/VEGFR2 and its induced PI3k/AKT/mTOR signal pathway.

## Figures and Tables

**Table 1:** Primers and probes which were used in real-time PCR.

Primer ID	Sequence
VEGFR2-forward	5′-CCA CAT GGT CTC TCT GGT TG-3′
VEGFR2-reverse	5′-GGA GGG TTG GCA TAG ACT GT-3′
VEGFR2-probe	5′-TGG CAC CAT GCA GAC GCT GA-3′
VEGF-forward	5′-TAT CTT CAA GCC GTC CTG TG-3′
VEGF-reverse	5′-GAT CCG CAT GAT CTG CAT AG-3′
VEGF-probe	5′-ATC ATT GCA GCA GCC CGC AC-3′
GADPH-forward	5′-GTT ACC AGG GCT GCC TTC TC-3′
GADPH-reverse	5′-GGG TTT CCC GTT GAT GAC C-3′
GADPH-probe	5′-AAC GGC ACA GTC AAG GCT GAG AAT G-3′

**Table 2:** Primary antibodies which were used in Western blotting.

Antibody	Supplier	Cat. number	Dilution ratio
anti-PI3K	Cell Signaling Technology	#4257	1 : 1000
anti-pPI3K	Cell Signaling Technology	#4228	1 : 500
anti-pAKT	Cell Signaling Technology	#4060	1 : 1000
anti-AKT	Cell Signaling Technology	#4691	1 : 1000
anti-pmTOR	Cell Signaling Technology	#5536	1 : 1000
anti-mTOR	Cell Signaling Technology	#2983	1 : 1000
